# Gliflozins, Erythropoietin, and Erythrocytosis: Is It Renal Normoxia- or Hypoxia-Driven?

**DOI:** 10.3390/jcm12144871

**Published:** 2023-07-24

**Authors:** Samuel N. Heyman, Zaid Abassi

**Affiliations:** 1 Department of Medicine, Hadassah Hebrew University Hospital, Mt. Scopus and Herzog Hospital, Jerusalem 9765422, Israel; 2 Department of Laboratory Medicine, Rambam Health Care Campus, Haifa 3109601, Israel; 3 Department of Physiology & Biophysics, The Rappaport Faculty of Medicine, Technion IIT, Haifa 3200003, Israel

**Keywords:** gliflozins, kidney, hypoxia, erythropoietin, erythrocytosis

## Abstract

The introduction of gliflozins in the management of type 2 diabetes mellitus leads to a better control of hyperglycemia, obesity, hypertension, dyslipidemia, and fluid retention. Most importantly, it also improves renal survival and reduces major cardiovascular events and mortality. Gliflozins were also found to induce erythropoietin (EPO) synthesis, leading to reticulocytosis and erythropoiesis. The mechanism(s) by which gliflozins induce erythropoiesis is a matter of debate. Although the canonical pathway of triggering EPO synthesis is through renal tissue hypoxia, it has been suggested that improved renal oxygenation may facilitate EPO synthesis via non-canonical routes. The latter proposes that the recovery of peritubular interstitial fibroblasts producing erythropoietin (EPO) is responsible for enhanced erythropoiesis. According to this hypothesis, enhanced glucose/sodium re-uptake by proximal tubules in uncontrolled diabetes generates cortical hypoxia, with injury to these cells. Once transport workload declines with the use of SGLT2i, they recover and regain their capacity to produce EPO. In this short communication, we argue that this hypothesis may be wrong and propose that gliflozins likely induce EPO through the documented intensification of renal hypoxia at the corticomedullary junction, related to the translocation of tubular transport from cortical segments to medullary thick ascending limbs. We propose that gliflozins, through intensified hypoxia in this region, trigger local EPO synthesis in peritubular interstitial cells via the canonical pathway of blocking HIF-prolyl hydroxylases (that initiate HIF alpha degradation), with the consequent stabilization of HIF-2 signal and an apocrinic induction of EPO in these same cells.

Gliflozins have provided a breakthrough in the management of type 2 diabetes. In addition to facilitating normoglycemia, these SGLT2 inhibitors attenuate obesity, hypertension, dyslipidemia, and fluid retention, reduce cardiovascular morbidity, retard the progression of renal dysfunction, and improve survival [1]. The administration of gliflozins also triggers erythropoietin (EPO) production, with the consequent induction of reticulocytosis and erythrocytosis [2]. The mechanism(s) by which gliflozins induce erythropoiesis is a matter of debate that we address in this short communication. Although the canonical pathway for triggering EPO synthesis is through renal tissue hypoxia, it has been suggested that improved renal oxygenation may facilitate EPO synthesis via non-canonical routes. 

Diabetes-induced renal hypoxia [3,4], attributed to increased tubular transport workload and the generation of reactive oxygen species, is caused by a rise in glomerular filtration rate (GFR) and glycosuria-associated osmotic diuresis. It has been proposed that intensified renal hypoxia and oxidative stress act together to predispose diabetic kidney to acute kidney injury (AKI) and in the progression of chronic renal disease (CKD) [5,6]. Gliflozins resolve renal cortical hypoxia [7] by inhibiting proximal tubular oxygen consumption for the SGLT2-mediated transport of glucose and sodium at the proximal tubule, and by the restoration of altered tubulo-glomerular feedback [8,9] (Figure 1). It has been further suggested that the amelioration of renal hypoxia may play a role in renal protection provided by these agents.

The renal production of EPO takes place in fibroblast-like interstitial peritubular cells located in the deep cortex [10]. Some 6 years ago, Sano et al. proposed that intensified cortical hypoxia in the diabetic kidney suppresses the induction of EPO synthesis by these cells and suggested that the improved cortical oxygenation noted with gliflozins generates the increase in EPO by the rescue of EPO-producing interstitial cells subjected to hypoxic and oxidative injury [11]. They further extended this narrative, 3 years later, in *Circulation*, suggesting that cytokines and oxidative stress in proximal tubular cells transform peritubular fibroblasts into myofibroblasts that lack the capability to express EPO [12]. Their concept is based on observations in mice, where damage to the proximal tubular epithelial cells induced by diphtheria toxin led to trans-differentiation of EPO-producing peritubular fibroblasts into myofibroblasts, which lose the capacity to produce EPO and generate fibrogenic molecules instead [13]. In line with Sano’s hypothesis, we have also noted that the antioxidant tempol intensifies HIF-1α and HIF-2α signals in tubular cells and in EPO-producing interstitial cells, respectively, by means unrelated to ambient oxygenation [4]. This indeed suggests that reducing oxidative stress might improve the capacity to generate HIF and HIF-dependent EPO synthesis. Additionally, Farsijani and colleagues documented cortical epithelial–interstitial cell cross-talk, where the non-hypoxic manipulation stabilizing HIF-α signal in tubular cells suppresses the population of EPO-producing interstitial cells [14]. Altogether, Sano’s hypothesis, which links erythropoiesis to the gliflozin-mediated restoration of cortical oxygenation (Figure 1, bottom, left-hand side), has been adopted and quoted in in-depth reviews about gliflozins recently published in the *New England Journal of Medicine* [1] and in the *European Heart Journal* [15]. 

We argue that this hypothesis is likely wrong in the setup of diabetes managed by gliflozins and contradicts what is known about the canonical regulation of EPO synthesis. First, there is no evidence for proximal tubular and cortical interstitial cell injury related to hypoxia in the diabetic kidney. Tubular rather than interstitial cells are prone to hypoxic injury, reflecting tubular cell transport-related oxygen expenditure in the diabetic kidney. However, uncontrolled diabetes is not enough to exert evident cell injury and additional perturbations are needed to exert hypoxic tubular injury [5]. Moreover, hypoxia, not normoxia, stimulates EPO synthesis by hypoxia-inducible factors (HIFs). HIFs are ubiquitous master regulators of the transcription of numerous genes involved in cell metabolism, proliferation, and survival, including EPO [16]. HIFs are heterodimers consisting of α and β subunits. HIF complexes formed in the cytoplasm undergo nuclear translocation and bind to the hypoxia-response elements promoting gene expression. Tissue oxygenation controls HIF signals through the regulation of the proteasomal degradation of continuously formed α subunits. HIF-α degradation is initiated by oxygen-sensitive HIF prolyl hydroxylases (PHDs), with the subsequent proteasomal degradation chaperoned by von Hippel–Lindau protein (VHL). Hypoxia blocks PHDs, leading to the cytoplasmic accumulation of HIF-α subunits, permeating its binding to β subunits, with the formed heterodimers undergoing nuclear translocation and initiating gene transcription, including EPO [16]. Notably, germline mutations in the genes encoding VHL, HIF-2α, and PHDs cause hereditary erythrocytosis, underscoring the canonical role of HIF-2α in the hypoxia-mediated regulation of EPO synthesis [17]. Most importantly, novel inhibitors of PHDs, specifically PHD2 inhibitors, markedly intensify the nuclear expression of HIF-2α in EPO-producing peritubular cells located in the deep cortex [10], and are now approved for use as EPO inducers in patients with advanced chronic kidney disease [18]. 

We argue that the hypothesis generated by Sano et al. [11,12], and adopted by others as an established fact, is conceivably wrong. First, cortical hypoxia, noted in diabetic animals, along with increased HIF-2 expression in peritubular interstitial cells, disappear following the administration of insulin [4]. This intervention attenuates renal hypoxia but does not lead to erythrocytosis, contradicting Sano’s hypothesis. Furthermore, there is unequivocal evidence that links gliflozins to renal hypoxia and to EPO synthesis. The administration of non-selective SGLT inhibitors indeed improves cortical oxygenation due to diminished transport activity along the proximal tubules. However, at the same time, renal outer medullary oxygenation declines [7], since sodium delivery to the distal nephron increases, with enhanced transport by medullary thick ascending limbs, which ascend along medullary rays and reach the cortex at the macula densa [9] (Figure 1). Our group has previously underscored the delicate balance of outer medullary oxygenation: limited regional blood flow and oxygen availability through the vasa recta puts this region on the verge of hypoxic injury, which is predominantly governed by the degree of oxygen expenditure for tubular transport [19,20]. Thus, conceivably, gliflozin-induced enhanced sodium delivery to medullary thick ascending limbs increases regional oxygen consumption for tubular transport and intensifies hypoxia in the interstitial cells that reside at the cortico-medullary junction and along cortical medullary rays. This would consequently generate HIF stabilization, triggering EPO synthesis (Figure 1, bottom, right-hand side). 

Finally, a recent large clinical trial has directly addressed the controversy, looking at the effect of semaglutide, empagliflozin, or their combination on hematocrit and on renal parenchymal oxygenation, determined by blood oxygen level-dependent (BOLD) MRI. Empagliflozin (but not semaglutide) increased hematocrit in parallel with declining medullary oxygenation, while cortical oxygenation remained unchanged [21]. These findings provide additional compelling evidence favoring our hypothesis. 

Thus, our explanation, based on well-established physiologic concepts regarding the canonical induction of EPO through intensified hypoxia at the corticomedullary junction, is at least as feasible as Sano’s hypothesis, linking erythrocytosis induced by gliflozins to the restoration of cortical normoxia. Regretfully, our comments on Sano’s theory six years ago [22] were ignored in their subsequent presentation of the same hypothesis [12]. This has unfortunately led prominent cardiologists, unfamiliar with renal physiology and with the regulation of EPO synthesis, to adopt Sano’s theory as an established unequivocal fact and to further distribute this plausible misconception in their recent review articles [1,15]. 

In summary, we propose that intensified hypoxia at the cortico-medullary junction, rather than improved cortical oxygenation, forms the canonical physiologic basis for the gliflozin-mediated increase in EPO synthesis and erythrocytosis. However, studies by Farsijani and colleagues [14] illustrate that EPO synthesis may be further facilitated by the restoration of the synthetic capacity of interstitial peritubular cells via a non-canonical pathway through the amelioration of cortical hypoxia. 

## Figures and Tables

**Figure 1:**
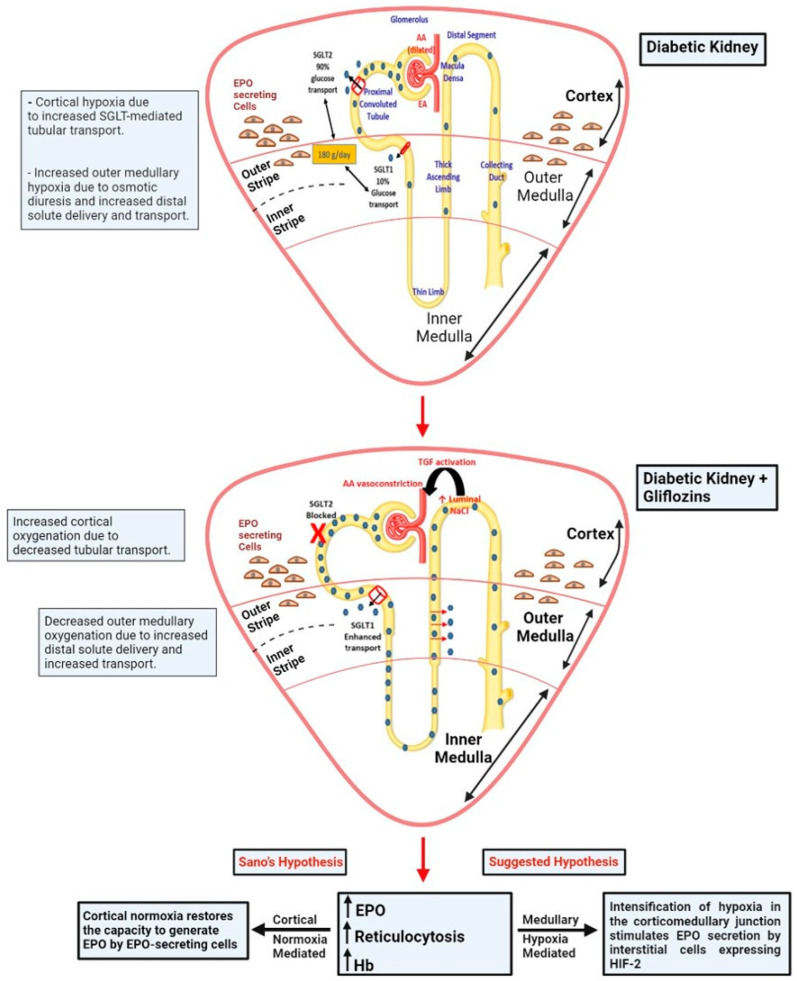
A scheme illustrating possible mechanisms involved in gliflozin-associated erythrocytosis. Diabetes induces renal cortical hypoxia and intensifies medullary physiologic hypoxia. Animal studies show that gliflozins restore cortical oxygenation, likely through the inhibition of energy expenditure for SGLT-2-mediated tubular transport. At the same time, outer medullary hypoxia is intensified, conceivably due to increased solute delivery to the distal nephron, enhancing tubular transport in medullary thick limbs. Gliflozins increase EPO synthesis and induce reticulocytosis and erythrocytosis. Peritubular fibroblast-like interstitial cells at the deep cortex and corticicomedullary junction generate renal EPO. The scheme shows that gliflozin-associated intensified EPO synthesis may reflect the enhanced HIF-2-mediated EPO synthesis in response to intensified medullary hypoxia (a canonical pathway). A non-canonical pathway may also exist (Sano’s hypothesis), whereby the restoration of cortical hypoxia in the diabetic kidney reestablishes the capacity of the peritubular interstitial cells to generate EPO (a non-canonical pathway).

## Data Availability

The data presented in this study are available upon request from the corresponding author.
